# Low levels of cathepsin D are associated with a poor prognosis in endometrial cancer

**DOI:** 10.1038/sj.bjc.6690090

**Published:** 1999-02

**Authors:** O Falcón, R Chirino, L León, A López-Bonilla, S Torres, L Fernández, J A García-Hernández, P F Valerón, J C Díaz-Chico

**Affiliations:** Department of Obstetrics and Gynaecology, Hospital Materno Infantil de Las Palmas de GC, Canary Islands, Spain; Department of Cellular and Molecular Endocrinology, Faculties of Medicine and Veterinary Science, University of Las Palmas de GC, Canary Islands, Spain; Clinical Research Unit, Complejo Hospitalario Materno Insular, Las Palmas de GC, Canary Islands, E-35080, Spain; Department of Pathology, Hospital Materno Infantil de Las Palmas de GC, Canary Islands, Spain; Department of Pharmacology, Faculties of Medicine and Veterinary Science, University of Las Palmas de GC, Canary Islands, Spain

**Keywords:** cathepsin D, prognosis, endometrial cancer

## Abstract

Total cytosolic cathepsin D (Cat D) levels were estimated by an immunoradiometric assay in a series of 156 consecutive patients with surgical stages I–III primary endometrial adenocarcinoma. Simultaneously, the tissue content of both oestrogen (ER) and progesterone (PR) receptors, and p185^HER-2/neu^, DNA content (ploidy), and the fraction of S-phase cells (S-phase) were also estimated. Tumoral Cat D content ranged from 0 to 243 pmol mg^−1^ protein (median 44 pmol mg^−1^ protein) and was not associated with any of the established clinicopathological and biological prognostic variables, with the exception of a weak positive correlation with the tumoral p185^HER-2/neu^ levels. Univariable analysis performed on a subset of 97 patients, followed for a minimum of 2 years or until death, showed that patient age at diagnosis, high histological grade, advanced surgical stage, vascular invasion, positive peritoneal cytology, low levels of Cat D, negative ER and PR status, aneuploidy, and high S-phase were predictive of the presence of persistent or recurrent disease. However, multivariable analysis revealed that only histological grade, surgical stage, Cat D and PR were significantly associated with the patient's outcome. From these findings, we conclude that Cat D is an independent prognostic factor in endometrial adenocarcinoma, its low levels being associated with a worse clinical outcome. © 1999 Cancer Research Campaign


*British Journal of Cancer* (1999) **79**(3/4), 570–576
© 1999 Cancer Research Campaign
Article no. bjoc.1998.0090

### Low levels of cathepsin D are associated with a poor prognosis in endometrial cancer

**O Falcón[1], R Chirino[2,3], L León[4], A López-Bonilla[1], S Torres[2], L Fernández[3,5], JA García-Hernández[1], PF Valerón[2] and JC Díaz-Chico[2,3]**

[1]Department of Obstetrics and Gynaecology, Hospital Materno Infantil de Las Palmas de GC, Canary Islands, Spain; [2]Department of Cellular and Molecular Endocrinology, Faculties of Medicine and Veterinary Science, University of Las Palmas de GC, Canary Islands, Spain; [3]Clinical Research Unit, Complejo Hospitalario Materno Insular, PO Box 550, E-35080 Las Palmas de GC, Canary Islands, Spain; [4]Department of Pathology, Hospital Materno Infantil de Las Palmas de GC, Canary Islands, Spain; [5]Department of Pharmacology, Faculties of Medicine and Veterinary Science, University of Las Palmas de GC, Canary Islands, Spain

**Summary** Total cytosolic cathepsin D (Cat D) levels were estimated by an immunoradiometric assay in a series of 156 consecutive patients with surgical stages I–III primary endometrial adenocarcinoma. Simultaneously, the tissue content of both oestrogen (ER) and progesterone (PR) receptors, and $p185^{HER\text{-}2/neu}$, DNA content (ploidy), and the fraction of S-phase cells (S-phase) were also estimated. Tumoral Cat D content ranged from 0 to 243 pmol $mg^{-1}$ protein (median 44 pmol $mg^{-1}$ protein) and was not associated with any of the established clinicopathological and biological prognostic variables, with the exception of a weak positive correlation with the tumoral $p185^{HER\text{-}2/neu}$ levels. Univariable analysis performed on a subset of 97 patients, followed for a minimum of 2 years or until death, showed that patient age at diagnosis, high histological grade, advanced surgical stage, vascular invasion, positive peritoneal cytology, low levels of Cat D, negative ER and PR status, aneuploidy, and high S-phase were predictive of the presence of persistent or recurrent disease. However, multivariable analysis revealed that only histological grade, surgical stage, Cat D and PR were significantly associated with the patient's outcome. From these findings, we conclude that Cat D is an independent prognostic factor in endometrial adenocarcinoma, its low levels being associated with a worse clinical outcome.

**Keywords:** cathepsin D; prognosis; endometrial cancer

In developed countries, endometrial cancer is the most common female pelvic malignancy. Although most of these cancers (75%) are detected at an early stage and present a favourable clinical outcome, a significant number of patients (10–15%) with localized disease develop recurrence and distant metastases and die of cancer (Malkasian et al, 1980; Lotocki et al, 1983). Several clinicopathological features are currently used as prognostic indicators, including histological type, histological grade, depth of myometrial invasion, surgical stage of disease, vascular invasion, peritoneal cytology, cervical extension, lymph node involvement, and the presence of metastatic disease (Christopherson et al, 1983; Creasman et al, 1987; Morrow et al, 1991; Barakat et al, 1997). The identification of new prognostic factors, more closely related to tumour biology, would be of great interest for their potential ability to discriminate between patients with low and high risk of recurrence, and even for treatment planning. In this respect, oestrogen (ER) and progesterone (PR) receptors (Creasman, 1993), DNA content (ploidy) (Newbury et al, 1990), percentage of cells in the S-phase of the cell cycle (S-phase) (Rosenberg et al, 1989), and HER-2/neu oncogene (Hetzel et al, 1992) have been extensively studied in the past years.

*Received 16 January 1998*
*Revised 22 May 1998*
*Accepted 15 June 1998*

*Correspondence to:* JC Díaz-Chico, Department of Cellular and Molecular Endocrinology, Health Science Centre, University of Las Palmas de Gran Canaria, PO Box 550, E-35080 Las Palmas de Gran Canaria, Canary Islands, Spain

Tumour invasion and metastasis are complex processes in which proteases that promote the breakdown of basement membrane and extracellular matrix play a central role, cathepsin D (Cat D) being one of the most studied. Cat D is an acidic aspartil lysosomal endopeptidase produced as a 52-kDa precursor, which is then processed to a 48-kDa intermediate, and then to a 34-kDa mature stable form (Caponi et al, 1986). In human breast cancer cells, altered normal sorting and maturation resulted in a marked increase in Cat D secretion (Caponi et al, 1989). Besides its enzymatic activity, Cat D is a mitogen, acting through its binding to the insulin-like growth factor type II receptor (Mathieu et al, 1990). Cat D expression is controlled by oestrogen and growth factors in human breast cancer cells (Westley et al, 1980; Rochefort et al, 1990). However, in both human endometrial cancer cell lines (Touitou et al, 1989), rat uterus (Elangovan et al, 1980), and normal human endometrium (Maudelonde et al, 1990), Cat D expression is under regulation by progesterone and growth factors, but not by oestrogen.

The usefulness of Cat D as a prognostic factor has been extensively studied in breast cancer in which high levels of the protease have been associated with a worse prognosis either in whole tumour series or in node-negative or node-positive patients (Spyratos et al, 1989; Thorpe et al, 1989; Tandon et al, 1990; Foekens et al, 1993; Ferrandina et al, 1997), although discrepant findings have also been reported (Ravdin et al, 1994, 1997). In

**Table 1** Distribution of cathepsin D levels according to clinicopathological parameters

| Parameter | *n* | Median (pmol mg$^{-1}$ protein) | Range (pmol mg$^{-1}$ protein) | *P*-value | Number of cases (%) <40 pmol mg$^{-1}$ protein | *P*-value |
|---|---|---|---|---|---|---|
| Age at diagnosis (years) | | | | | | |
| <65 | 75 | 44 | 4–176 | | 27 (36.0) | |
| ≥65 | 81 | 44 | 0–243 | ns | 33 (40.7) | ns |
| Menopausal status | | | | | | |
| Pre/peri | 10 | 53 | 14–176 | | 4 (40.0) | |
| Post | 146 | 44 | 0–243 | ns | 56 (38.4) | ns |
| Histological type | | | | | | |
| Endometrioid | 139 | 44 | 4–243 | | 52 (37.4) | |
| Other | 17 | 42 | 0–114 | ns | 8 (47.1) | ns |
| Histological grade | | | | | | |
| 1 | 80 | 53 | 4–185 | | 25 (33.3) | |
| 2 | 42 | 43 | 20–243 | | 18 (42.9) | |
| 3 | 34 | 47 | 0–173 | ns | 17 (44.7) | ns |
| Myometrial invasion | | | | | | |
| None–<50% | 117 | 49 | 4–185 | | 42 (36.5) | |
| >50% | 39 | 42 | 0–243 | ns | 18 (43.9) | ns |
| Stage | | | | | | |
| I | 114 | 47 | 4–185 | | 41 (35.7) | |
| II | 23 | 45 | 14–243 | | 10 (41.7) | |
| III | 19 | 44 | 0–173 | ns | 9 (52.9) | ns |
| Vascular invasion | | | | | | |
| No | 121 | 44 | 4–243 | | 44 (36.4) | |
| Yes | 35 | 55 | 0–142 | ns | 16 (45.7) | ns |
| Peritoneal cytology | | | | | | |
| Negative | 115 | 44 | 8–243 | | 42 (36.5) | |
| Positive | 11 | 35 | 0–142 | ns | 6 (54.5) | ns |

ns, non-significant.

contrast, there are few data on Cat D in endometrial cancer, and only one report deals with its prognostic value (Lösch et al, 1996).

In the present study, we examined the relationship between Cat D and established clinicopathological prognostic parameters and biological predictors in a series of 156 patients with primary endometrial cancer. The prognostic value of Cat D was also studied in a subset of 97 patients followed for at least 2 years or until death. Our results suggest that a low tumoral Cat D content is predictive of persistent or recurrent disease.

### MATERIALS AND METHODS

### Patients

This study was performed on 156 consecutive patients diagnosed with surgical stage I–III primary endometrial cancer between June 1990 and June 1997, and treated at the Department of Obstetrics and Gynaecology at the Hospital Materno Infantil in Las Palmas (Canary Islands, Spain). None of the patients had received substitutive hormonal therapy for at least 3 months before diagnosis. All patients underwent exploratory laparotomy, total hysterectomy and bilateral salpingo-oophorectomy. Radiation therapy after surgery was administered to all patients in stages II and III, and to patients in stage I with risk factors such as serous and clear cell histological types, deep myometrial invasion, and high histological grade. The treatment consisted of irradiation of either the vaginal cuff (45–60 Gy) ($n = 8$), the whole pelvis (50 Gy) ($n = 5$), or both ($n = 62$). Additionally, two patients received irradiation in the whole pelvis and in the para-aortic nodes (45 Gy). Complete clinical information was obtained for all patients. This included age at diagnosis, menopausal status, histological type, histological grade, depth of myometrial invasion, surgical stage according to the International Federation of Gynaecology and Obstetrics (FIGO) classification, vascular invasion, and presence of metastatic disease. Information on peritoneal affection was available in 126 patients, but that on lymph node involvement was very limited because lymphadenectomy was performed in only 16 cases.

### Tissue fractionation

Tumour specimens were promptly frozen in liquid nitrogen after surgery and then kept at –70°C until assay. The percentage of tumoral tissue (cancer cell plus stroma) present in each specimen was almost 100% in most cases (selected under macroscopic inspection by the pathologist). Samples (100–200 mg) were homogenized in ten volumes of buffer containing 10 mM tris-HCl, pH 7.4, 1.5 mM disodium EDTA, 10 mM sodium molybdate, 0.1% monothioglycerol, 1 mM phenylmethylsulphonyl fluoride, 1 μg ml$^{-1}$ aprotinin, 1 mM sodium orthovanadate and 10% glycerol. Aliquots of homogenates were used to measure the total cellular p185$^{HER\text{-}2/neu}$ (p185) content. The rest of the homogenates were centrifuged at 1000 ***g*** for 15 min at 4°C, and the supernatants were centrifuged again at 105 000 ***g*** for 1 h at 4°C to obtain the cytosols.

© Cancer Research Campaign 1999 

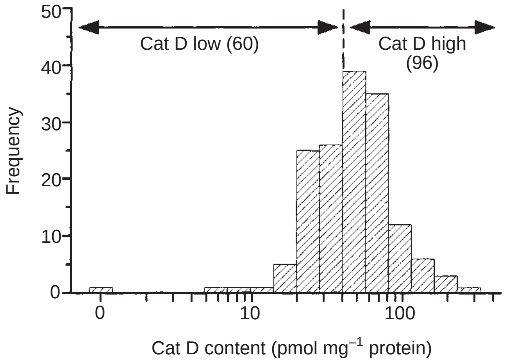

**Figure 1** Frequency distribution of cathepsin D values, logarithmically transformed, in 156 endometrial tumour cytosols, divided at the selected cut-off point of 40 pmol mg$^{-1}$ protein. Numbers in parentheses indicate the number of tumours

### Assays

Cat D was measured in the cytosol using a solid phase two-site radiometric immunoassay (Elsa-Cath-D; Cis biointernational, Gif-sur-Yvette, France). Briefly, cytosols were diluted to a protein concentration of 1 mg ml$^{-1}$ and the assay was performed in duplicate after a further 81-fold dilution. The best cut-off level to consider that a given specimen contained a high content of Cat D was 40 pmol mg$^{-1}$ protein (see below).

For the measurement of ER and PR, cytosols were diluted to 1 mg protein ml$^{-1}$ with 0.1% bovine serum albumin (BSA) and were incubated overnight in duplicate at 0–4°C with either 5 nM of [$^3$H]-oestradiol or 10 nM of [$^3$H]R5020 (NEN, Boston, MA, USA), in the presence or absence of a 200-fold excess of unlabelled diethylstilboestrol or R5020, respectively, to correct for non-specific binding. At the end of the incubation period, the unbound steroid was removed by dextran-coated charcoal treatment (0.05% dextran T-70, 0.5% activated charcoal). Tumours were classified as ER or PR positive if the content of ER and PR was equal to or greater than 10 and 30 fmol mg$^{-1}$ protein respectively.

The total cellular p185 content was measured in the homogenates from tumour samples by a commercial enzyme-linked immunosorbent assay (ELISA) (human neu-oncoprotein ELISA; Oncogene Science, Uniondale, NY, USA). Aliquots of 50 µl of the tissue homogenates were incubated for 5 min at room temperature with 10 µl of antigen-extraction agent and centrifuged at 14 000 ***g*** for 10 min at 4°C. The protein concentration of supernatant was diluted to 10 µg ml$^{-1}$, or further if necessary, and the assay was performed in duplicate according to the instructions given by the manufacturer. A level of p185 equal to or higher than 260 fmol mg$^{-1}$ protein, previously validated by us in breast cancer (Valerón et al, 1996), was used to consider that a given tumour overexpressed the oncoprotein.

The protein concentration was estimated by the Bradford method (Bio-Rad, Richmond, CA, USA) using BSA as a standard.

For flow cytometry analysis, fine-needle aspiration of tissue samples was used for initial mechanical disaggregation. The aspirates were flushed with buffer containing 3.4 mM trisodium citrate, pH 7.6, 0.1% Nonidet P-40, 1.5 mM spermine tetrahydrochloride and 0.5 mM Tris. The suspensions were then clarified by filtering through a 50-µm nylon mesh and centrifuged at 200 ***g*** for 5 min. Pellets were resuspended in 100 µl of buffer and treated according to the method of Vindelov and Christensen (1990). Stained nuclei were analysed on an EPICS XL-MCL cytometer (Coulter Corporation, Hialeah, FL, USA). Quality control of the flow cytometer was carried out using DNA-Check Epics Alignment Fluorospheres (Coulter). Peak integrated red fluorescence, gated along the diagonal, was used to analyse 20 000 nuclei, and data were stored in list mode. Half-peak coefficients of variation were always lower than 5%. Generated histograms were analysed for cell cycle compartments and background debris subtraction using the Multicycle software for cell cycle analysis (Phoenix Flow Systems, San Diego, CA, USA). In accordance with previous studies (Lukes et al, 1994), tumours with a DNA index equal to or greater than 1.25 were considered aneuploid. The best cut-off point to discriminate between high and low S-phase was 11% (see below).

**Table 2** Levels of continuous biological prognostic indicators in 156 endometrial tumours

| Prognostic variable | Mean | s.d. | Median | Minimum | Maximum |
|---|---|---|---|---|---|
| Cat D | 53 | 36 | 44 | 0 | 243 |
| ER | 106 | 113 | 72 | 0 | 737 |
| PR | 442 | 472 | 281 | 0 | 2069 |
| p185 | 219 | 376 | 159 | 12 | 4030 |
| S-phase | 6.8 | 5.2 | 5.1 | 0.7 | 24.7 |

### Follow-up

All patients were routinely examined every 3 months for the first 2 years and then every 6 months. A subset of 97 patients, who were followed for at least 2 years or until death, was selected to study the prognostic value of the variables analysed. Time to event was defined as the period of time from diagnosis to either the date of recurrence for patients with a disease-free interval after surgery or the date of surgery for patients who never became free of disease. For the whole subset, the median follow-up time was 42 months (range 0.2–91 months). There were 22 patients with either recurrent ($n$ = 17) or persistent ($n$ = 5) disease. Their median follow-up time was 14 months (range 0.2–83 months). Among these patients, 15 (68.2%) died of endometrial cancer, including five out of five (100%) patients with persistent disease and 10 out of 17 (58.8%) patients with recurrent disease. The remaining 75 patients without recurrent or persistent disease were alive at the last follow-up. The median follow-up time of these patients was 49 months (range 24–91 months).

### Statistical analysis

Correlations between Cat D and steroid receptors, p185 and S-phase were performed by Spearman's rank correlation coefficient. The non-parametric Mann–Whitney or Kruskal–Wallis tests were used for comparison between the medians of two or more groups respectively. The relationship between Cat D status and that of other prognostic variables was examined in contingency tables using the chi-squared or Fisher's exact tests. Cat D and S-phase were dichotomized using the minimum $P$-value method by selecting the best cut-off value that allowed the maximal separation between groups at low and high risk for persistence or relapse. To confirm the statistical significance of the selected value, the levels of the studied variables were logarithmically transformed

© Cancer Research Campaign 1999

**Table 3** Distribution of cathepsin D levels according to biological parameters

| Parameter | n | Median (pmol $mg^{-1}$ protein) | Range (pmol $mg^{-1}$ protein) | P-value | Number of cases (%) <40 pmol $mg^{-1}$ protein | P-value |
|---|---|---|---|---|---|---|
| ER | | | | | | |
| Negative | 28 | 52 | 0–176 | | 12 (42.9) | |
| Positive | 128 | 44 | 4–243 | ns | 48 (37.5) | ns |
| PR | | | | | | |
| Negative | 25 | 38 | 0–97 | | 14 (56.0) | |
| Positive | 131 | 45 | 4–243 | ns | 46 (35.1) | ns |
| p185 | | | | | | |
| Low | 128 | 44 | 0–243 | | 53 (41.4) | |
| High | 28 | 53 | 20–157 | ns | 7 (25.0) | ns |
| Ploidy | | | | | | |
| Diploid | 131 | 44 | 4–243 | | 51 (38.9) | |
| Aneuploid | 25 | 47 | 0–142 | ns | 9 (36.0) | ns |
| S-phase | | | | | | |
| Low | 128 | 44 | 0–243 | | 58 (47.6) | |
| High | 28 | 48 | 14–176 | ns | 19 (55.9) | ns |

ns, non-significant. Cut-off levels of variables were as follows: ER, 10 fmol $mg^{-1}$ protein; PR, 30 fmol $mg^{-1}$ protein; p185, 260 fmol $mg^{-1}$ protein; S-phase, 11%.

and the variable treated as a continuous covariate in a Cox model, as proposed by Altman et al (1994). *P*-values for Cat D and S-phase in the Cox analysis were 0.0062 and 0.0015 respectively. Univariate survival analysis was performed using the log-rank test in association with Kaplan–Meier analysis. The Cox proportional hazards model, with forward step-wise selection of variables, was applied for multivariable analysis. The likelihood ratio test was used to test for variable selection. In these studies, all variables were considered as binary, including age at diagnosis (≥ 65 years vs <65 years), grade (3 vs 1 or 2), stage (II or III vs I), and treatment (surgery plus radiotherapy vs surgery alone). The two-sided test of significance with an alpha of 0.05 was used in all analyses. Statistical analyses were performed using the SPSS for Windows (version 6.1.3) statistical software (SPSS, Chicago, IL, USA).

### RESULTS

### Characteristics of the tumours

The characteristics of our tumour series are listed in Table 1. The mean and median age of the patients was 65 years (range 29–93 years). Most of the patients were post-menopausal (93.6%), and the predominant histological type was endometrioid (89.1%). Among the 17 tumours of non-endometrioid histological type, five were serous and 12 clear cell. Half of the tumours were well differentiated (51.3%) and two-thirds were confined to the endometrium or invaded less than half of the myometrium. Surgical stage I was predominant (73.1%), vascular space involvement existed in 22.4% of the patients, and 8.7% of cases had a positive peritoneal cytology.

The Cat D content of the tumours followed a gaussian distribution when the values were logarithmically transformed (Figure 1). According to the selected cut-off point, 38.5% of tumours had a low Cat D level, whereas 61.5% had a high content. The descriptive statistic of all quantitatively analysed variables is shown in Table 2. The number of positive specimens for ER and PR was 82.1% and 84% respectively. The p185 content was elevated in 17.9% of cases, and 17.9% of tumours had a high S-phase. Furthermore, 16% of tumours were aneuploid, considering a DNA index of 1.25 as the cut-off point.

**Table 4** Univariate survival analysis of risk factors for persistent or recurrent endometrial cancer

| Variable | P-value |
|---|---|
| Age at diagnosis | 0.045 |
| Histological type | 0.24 |
| Histological grade | 0.0000 |
| Myometrial invasion | 0.38 |
| Stage | 0.0000 |
| Vascular invasion | 0.0028 |
| Peritoneal cytology | 0.0000 |
| Cathepsin D | 0.0015 |
| Oestrogen receptor | 0.027 |
| Progesterone receptor | 0.0000 |
| p185 | 0.56 |
| Ploidy | 0.0012 |
| S-phase | 0.0000 |
| Treatment | 0.47 |

Cut-off levels for biological variables as in Table 3.

### Relationship between Cat D content and clinicopathological or biological parameters

Table 1 also shows the relationship between Cat D levels and the clinicopathological characteristics of patients. There was no association between the tumoral Cat D content, considered either as continuous or categorical variable, and any of the established prognostic parameters.

Spearman correlation analyses between Cat D and steroid receptors, p185 and S-phase showed that Cat D did not correlate to any of the quantitatively analysed variables, with the exception of a weak positive correlation with p185 ($r = 0.2$; $P = 0.01$). Similarly, there was no association between Cat D content, considered either as a continuous or categorical variable, and the rest of the biological variables analysed (Table 3).

### Survival analysis

Results of the univariable survival analysis are shown in Table 4. Age at diagnosis, high histological grade, advanced surgical stage,

© Cancer Research Campaign 1999

**Table 5** Multivariate survival analysis of risk factors for persistent or recurrent endometrial cancer

| Variable | Wald chi-squared | RR (95% CI)[a] | *P*-value |
|---|---|---|---|
| Histological grade | | | |
| 3 vs 1 or 2 | 3.73 | 1.6 (0.99–2.58) | 0.054 |
| Stage | | | |
| II or III vs I | 10.71 | 2.25 (1.38–3.66) | 0.0011 |
| Cathepsin D | | | |
| Low vs High | 9.18 | 2.19 (1.32–3.65) | 0.0024 |
| Progesterone receptor | | | |
| Negative vs positive | 5.58 | 1.9 (1.12–3.23) | 0.018 |

[a]RR (95% CI), estimated risk ratio (95% confidence interval). For selected cut-off points of biological variables see Table 3.

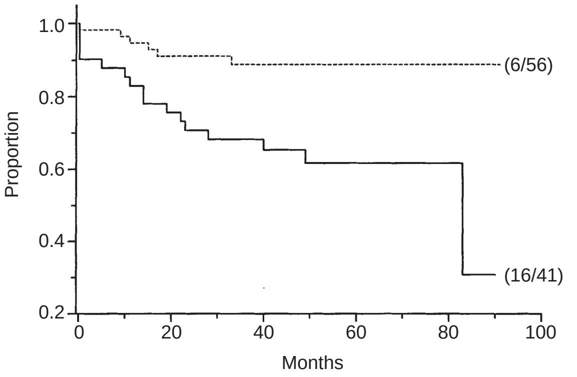

**Figure 2** Kaplan and Meier survival curve of patients stratified according to cathepsin D status defined by the cut-off value of 40 pmol mg$^{-1}$ protein (- - -) Cat D high; (—) Cat D low. Numbers in parentheses indicates the number of failures/total number of patients in each group. The difference was significant using the log-rank test. *P* = 0.0015

**Table 6** Survival rates by type of treatment and cathepsin D status

| Treatment | *n* | Cat D (%) | | *P*-value |
|---|---|---|---|---|
| | | Low | High | |
| Surgery | 54 | 35.7 | 89.9 | <0.05 |
| Surgery plus radiotherapy | 43 | 48.8 | 87 | <0.05 |

low levels of Cat D, negative ER and PR status, aneuploidy, high S-phase, vascular invasion and positive peritoneal cytology were positively associated with persistent or recurrent disease. The multivariate survival analysis (Table 5), performed without considering peritoneal cytology because of the small number of patients in which it was estimated ($n$ = 73), showed that histological grade, surgical stage, Cat D and PR were significantly associated with disease-free survival. Among them, Cat D had one of the higher risk ratios; thus, in a given patient with a low Cat D content, the hazard risk of persistent or recurrent disease was 2.19-fold higher than in those patients with a Cat D level above the cut-off point. The Kaplan and Meier curve for Cat D is depicted in Figure 2. Although 39% of patients with low Cat D levels had persistent or recurrent disease at the last follow-up, this circumstance existed in only 10.7% of those patients with a Cat D content above the cut-off point ($P$ = 0.0015). To discard the possibility that the treatment modality biased these results, a separate analysis was performed for patients treated with surgery alone, or with surgery and radiotherapy. In each subgroup, patients with a low Cat D content had a significantly worse outcome than patients with high Cat D (Table 6).

### DISCUSSION

The literature contains very little information about the role of Cat D in endometrial cancer. To date, only six articles deal with Cat D expression in this tumour type, and its prognostic significance has only been studied in one of them. These studies were performed on relatively small series, because only one group included more than 100 cases (Lösch et al, 1996). In one study, Cat D levels were quantified by ELISA (Maudelonde et al, 1990), and in three (Nazeer et al, 1992; Scambia et al, 1995; Sanfilippo et al, 1996) by an immunoradiometric (IRMA) procedure similar to that used by us. In another article, Cat D was detected by immunohistochemistry (IHC) (Lösch et al, 1996). The two procedures (IRMA and IHC) were compared in one article (Nazeer et al, 1994) using a patient series very similar to one in which Cat D concentration was determined by IRMA by the same group (Nazeer et al, 1992).

It has been suggested that the tumoral cytosolic Cat D values obtained by the immunoradiometric assay used in this work may be impaired by the relative contribution of non-tumoral cells (Isola et al, 1993), which makes the IHC method more specific. However, some authors have reported that total cytosolic Cat D correlates well with Cat D content in cancer cells (Roger et al, 1994), and that Cat D levels in cancer and stromal cells are directly correlated (Ravdin et al, 1994). In addition, the assessment of Cat D content by the immunoradiometric assay seems likely to give reliable and comparable interstudy results as assessed by the EORTC Receptor Study Group (Benraad et al, 1992).

Our results showed that Cat D is expressed in variable amounts in endometrial cancer, its levels being slightly lower than or similar to those reported by some authors in breast cancer (Foekens et al, 1993; Marsigliante et al, 1994; Gion et al, 1995; Valerón et al, 1997). Our data in endometrial cancer are similar to those published by Sanfilippo et al (1996) and higher than those reported by three other groups (Maudelonde et al, 1990; Nazeer et al, 1992; Scambia et al, 1995). The reasons underlying these divergent results are not clear because most of these authors used essentially the same methodology as that used by us.

We did not find an association between Cat D levels and those of the established clinicopathological prognostic factors, which

© Cancer Research Campaign 1999

agrees with data reported by Lösch et al (1996), and contrasts with other reports in which Cat D content was positively associated with histological type (Nazeer et al, 1992), myometrial invasion (Maudelonde et al, 1990; Nazeer et al, 1992), and grade of differentiation (Nazeer et al, 1992). Conversely, Scambia et al (1995) reported an inverse association between Cat D and myometrial invasion and stage. One possible explanation for these discrepancies could rely on the relatively small series which most of the cited studies used.

There is evidence that Cat D gene expression is increased by progesterone, but not by oestrogen, both in rat uterus (Elangovan et al, 1980) and in normal human endometrium (Maudelonde et al, 1990). We did not find any correlation or association between Cat D and steroid hormone receptors, either in the whole tumour series or in the subgroup of PR-positive tumours (data not shown). These results are in accordance with two previous reports (Maudelonde et al, 1990; Nazeer et al, 1992) and disagree with another in which a positive association between Cat D and ER/PR status was found (Scambia et al, 1995). Our data suggest that in endometrial cancer the hormone dependence of Cat D expression is lost or, alternatively, the role that growth factors play in its regulation are more important than that exerted by sex hormones.

There are no data in the literature regarding the relationship between Cat D and p185, ploidy and S-phase in endometrial cancer. We could not find any correlation or association between Cat D and ploidy and S-phase. Regarding p185, we observed a weak, although significant, positive correlation between these two variables, a finding that has also been described in breast cancer (Seshadri et al, 1994; Valerón et al, 1997).

To establish the prognostic value of a set of variables, a large series of patients followed for a long time is desirable. Our series did not completely fulfil these criteria, and for this reason we selected a subset of patients followed for at least 2 years or until death. Because the follow-up period was relatively short (median 46 months), we used survival analysis for persistent or recurrent disease, a clinical condition which is difficult to cure, instead of overall survival, which would require a more prolonged follow-up.

As expected, univariate survival analysis confirmed the prognostic value of known clinicopathological prognostic factors, including age at diagnosis, histological type, histological grade, surgical stage, vascular invasion and peritoneal cytology, which is in accordance with previous reports (Christopherson et al, 1983; Creasman et al, 1987; Morrow et al, 1991; Barakat et al, 1997). However, histological type and depth of myometrial invasion lacked significance. This analysis also showed the prognostic significance of Cat D, ER, PR, ploidy and S-phase, but not that of p185. Multivariate analysis led us to determine the significance of biological factors in relation to conventional clinicopathological prognostic parameters. Our results showed that a high grade of differentiation, advanced surgical stage, low levels of cathepsin D and PR negativity are predictive of poor outcome. Therefore, and contrary to what might be expected, Cat D seems to be a favourable prognostic factor in endometrial cancer. This finding contrasts with that reported by Lösch et al (1996) using immunohistochemistry to detect the protease. However, Scambia et al (1995) reported that high Cat D levels might be a good prognostic factor in endometrial cancer, although they could not obtain reliable survival analysis. As far as we know, this is the first time in which such an association has been described in endometrial cancer, although before us Henry et al (1990) found similar results in breast cancer. One could argue that high Cat D content reflects the functional integrity of the steroid hormone receptor pathway (Henry et al, 1990), and that high levels of Cat D, as well as PR-positivity, are predictive of good prognosis. However, in our study, we did not find any relationship between Cat D and these receptors. It may also be possible that the acidic conditions needed for the Cat D to be active (Morisset et al, 1986) do not arise in endometrial cancer, but this does not explain the favourable incidence on prognosis of low Cat D levels. The question, then, is open to future research.

### ACKNOWLEDGEMENTS

This work was supported by grants from the Gobierno Autónomo de Canarias and from the Fondo de Investigaciones Sanitarias (Ministry of Health, Spain). We thank Mr JF Rivero, Ms BP Díaz and Ms F Guillén for their technical assistance, Dr P Saavedra for the mathematical analysis of the data, and Ms S Cranfield for her help in the preparation of the manuscript.

### REFERENCES

Altman DG, Lausen B, Sauerbrei W and Schumacher M (1994) Dangers of using “optimal” cutpoints in the evaluation of prognostic factors. *J Natl Cancer Inst* **86**: 829–835

Barakat RR, Park RC, Grigsby PW, Muss HD and Norris H (1997) Corpus: epithelial tumors. In *Principles and Practice of Gynecologic Oncology*, 2nd edn. Hoskins WJ, Perez CA and Young RC (eds.), pp. 859–896. Lippincott-Raven: Philadelphia

Benraad ThJ, Geurts-Moespot A, Sala M, Piffanelli A, Ross A and Foekens JA (1992) Quality control of cathepsin D measurement by the EORTC Receptor Study Group. *Eur J Cancer* **28A**: 72–75

Capony F, Garcia J, Capdevielle C, Rougeot C, Ferrara P and Rochefort H (1986) Purification and first characterization of the secreted and cellular 52-kDa proteins regulated by estrogens in human breast cancer cells. *Eur J Biochem* **161**: 505–512

Capony F, Rougeot C, Montcourrier P, Cavailles V, Salazar G and Rochefort H (1989) Increased secretion, altered processing, and glycosilation of procathepsin D in human mammary cancer cells. *Cancer Res* **46**: 3904–3909

Christopherson WM, Connelly PJ and Alberhasky RC (1983) An analysis of prognosticators in patients with favorable subtypes and stage I disease. *Cancer* **51**: 1705–1709

Creasman WT (1993) Prognostic significance of hormone receptors in endometrial cancer. *Cancer* **71**: 1467–1470

Creasman WT, Morrow PC, Bundy BN, Homesley HD, Graham JE and Heller PB (1987) Surgical pathologic spread patterns of endometrial cancer: a Gynecologic Oncology Group Study. *Cancer* **60**: 2035–2041

Elangovan S and Moulton BC (1980) Progesterone and estrogen control of rates of synthesis of uterine cathepsin D. *J Biol Chem* **255**: 7474–7479

Ferrandina G, Scambia G, Bardelli F, Benedetti Panici P, Mancuso S and Messori A (1997) Relationship between cathepsin-D content and disease-free survival in node-negative breast cancer patients: a meta-analysis. *Br J Cancer* **76**: 661–666

Foekens JA, van Putten WLJ, Portengen H, de Koning HYWCM, Thirion B, Alexieva-Figusch J and Klijn JGM (1993) Prognostic value of pS2 and cathepsin D in 710 human primary breast tumors: multivariate analysis. *J Clin Oncol* **11**: 899–908

Gion M, Mione R, Dittadi R, Romanelli M, Pappagallo L, Capitanio G, Friede U, Barbazza R, Visonà A and Dante S (1995) Relationship between cathepsin D and other pathological and biological parameters in 1752 patients with primary breast cancer. *Eur J Cancer* **31A**: 671–677

Henry JA, McCarthy AL, Angus B, Westley BR, May FEB, Nicholson S, Cairns J, Harris AL and Horne CHW (1990) Prognostic significance of the oestrogen-regulated protein, cathepsin D, in breast cancer. An immunohistochemical study. *Cancer* **15**: 265–271

Hetzel DJ, Wilson TO, Keeney GL, Roche PC, Cha SS and Podratz KC (1992) HER-2/neu expression: a major prognostic factor in endometrial cancer. *Gynecol Oncol* **47**: 179–185

Isola J, Weitz S, Visakorpi T, Holli K, Shea R, Khabbez N and Kallioniemi OP (1993) Cathepsin D expression detected by immunohistochemistry has independent prognostic value in axillary node-negative breat cancer. *J Clin Oncol* **11**: 36–43

Lösch A, Kohlberger P, Gitsch G, Kaider A, Breitnecker G and Kainz Ch (1996) Lysosomal protease cathepsin D is a prognostic marker in endometrial cancer. *Br J Cancer* **73**: 1525–1528

Lotocki RJ, Copeland LJ, DePetrillo AD and Muirhead W (1983) Stage I endometrial adenocarcinoma: treatment results in 835 patients. *Am J Obstet Gynecol* **146**: 141–144

Lukes AS, Kohler MF, Pieper CF, Kerns BJ, Bentley R, Rodriguez GC, Soper JT, Clarke-Pearson DL, Bast Jr RC and Berchuck A (1994) Multivariable analysis of DNA ploidy, p53, and HER-2/*neu* as prognostic factors in endometrial cancer. *Cancer* **73**: 2380–2385

Malkasian GD, Annegars JF and Fountain KS (1980) Carcinoma of the endometrium: stage 1. *Am J Obstet Gynecol* **136**: 872–888

Marsigliante S, Biscozzo L, Correale M, Paradiso A, Leo G, Abbate I, Dragone CD and Storelli C (1994) Immunoradiometric detection of pS2 and total cathepsin D in primary breast cancer biopsies: their correlation with steroid receptors. *Br J Cancer* **69**: 550–554

Mathieu M, Rochefort H, Barenton B, Prebois C and Vignon F (1990) Interaction of the cathepsin D and IGF-II on the M6P/IGF-II receptor in human breast cancer cells and possible consequences on mitogenic activity of IGF-II. *Mol Endocrinol* **4**: 1327–1335

Maudelonde T, Martinez P, Brouillet JP, Laffargue F, Pages A and Rochefort H (1990) Cathepsin-D in human endometrium: induction by progesterone and potential value as a tumor marker. *J Clin Endocrinol Metab* **70**: 115–121

Morisset M, Capony F and Rochefort H (1986) The 52 kDa estrogen-induced protein secreted by MCF7 cells is a lysosomal acidic protease. *Biochem Biophys Res Commun* **138**: 102–109

Morrow CP, Bundy BN, Kurman RJ, Creasman WT, Heller P, Homesley HD and Graham JE (1991) Relationship between surgical–pathological risk factors and outcome in clinical stage I and II carcinoma of the endometrium. *Gynecol Oncol* **40**: 55–65

Nazeer T, Malfetano JH, Rosano TG and Ross JS (1992) Correlation of tumor cytosol cathepsin D with differentiation and invasiveness of endometrial adenocarcinoma. *J Clin Pathol* **97**: 764–769

Nazeer T, Church K, Amato C, Rosano TG, Malfetano, JH and Ross JS (1994) Comparative quantitative inmunohistochemical and immunoradiometric determinations of cathepsin D in endometrial adenocarcinoma: predictors of tumor agressiveness. *Mod Pathol* **7**: 469–474

Newbury R, Schuerch C, Goodspeed N, Fanning J, Glidewell O and Evans M (1990) DNA content as a prognostic factor in endometrial carcinoma. *Obstet Gynecol* **76**: 251–257

Ravdin PM, Tandon AK, Allred DC, Clark GM, Fuqua SAW, Hilsenbeck SH, Chamness GC and Osborne CK (1994) Cathepsin D by Western blotting and immunohistochemistry: failure to confirm correlations with prognosis in node-negative breast cancer. *J Clin Oncol* **12**: 467–474

Ravdin PM, de Moor CA, Hilsenbeck SG, Samoszuk MK, Vendely PM and Clark GM (1997) Lack of prognostic value of cathepsin D levels for predicting short term outcomes of breast cancer patients. *Cancer Lett* **116**: 177–183

Rochefort H, Capony F and Garcia M (1990) Cathepsin D in breast cancer: from molecular and cellular biology to clinical applications. *Cancer Cells* **2**: 383–387

Roger P, Montcourrier P, Maudelonde T, Brouillet JP, Pages A, Laffargue F and Rochefort H (1994) Cathepsin D immunostaining in paraffin-embedded breast cancer cells and macrophages: correlation with cytosolic assay. *Hum Pathol* **25**: 863–871

Rosenberg P, Wingren S, Simonsen E, Stal O, Risberg B and Nordenskjold B (1989) Flow cytometric measurements of DNA index and S-phase on paraffin embedded early stage endometrial cancer: an important prognostic indicator. *Gynecol Oncol* **35**: 50–54

Sanfilippo JS, Miseljic S, Yang AR, Doering DL, Shaheen RM and Wittliff (1996) Quantitative analyses of epidermal growth factor receptors, HER-2/neu oncoprotein and cathepsin D in nonmalignant and malignant uteri. *Cancer* **77**: 710–716

Scambia G, Benedetti Panici P, Ferrandina G, Distefano M, Romanini ME, Sica G and Mancuso S (1995) Significance of cathepsin-D expression in uterine tumors. *Eur J Cancer* **31A**: 1449–1454

Seshadri R, Horsfall DJ, Firgaira F, McCaul K, Setlur V, Chalmers AH, Yeo R, Ingram D, Dawkins H, Hahnel R and the South Australian Breast Cancer Study Group (1994) The relative prognostic significance of total cathepsin D and HER-2/neu oncogene amplification in breast cancer. *Int J Cancer* **56**: 61–65

Spyratos F, Brouillet JP, Defrenne A, Hacene K, Rouesse J, Maudelonde T, Brunet M, Andrieu C, Desplaces A and Rochefort H (1989) Cathepsin D: and independent prognostic factor for metastasis of breast cancer. *Lancet* **2**: 1115–1118

Tandon AK, Clark GM, Chamness GC, Chirgwin JM and McGuire WL (1990) Cathepsin D and prognosis in breast cancer. *N Engl J Med* **322**: 297–302

Thorpe S, Rochefort H and Garcia M (1989) Association between high concentration of Mr 52,000 cathepsin D and poor prognosis in primary human breast cancer. *Cancer Res* **49**: 6008–6014

Touitou I, Cavailles V, Garcia M, Defrenne A and Rochefort H (1989) Differential regulation of cathepsin D by sex steroid in mammary cancer and uterine cells. *Mol Cell Endocrinol* **66**: 231–238

Valerón PF, Chirino R, Fernández L, Torres S, Navarro D, Aguiar J, Cabrera JJ, Díaz-Chico BN and Díaz-Chico JC (1996) Validation of a differential PCR and an ELISA procedure in studying HER-2/neu status in breast cancer. *Int J Cancer* **65**: 129–133

Valerón PF, Chirino R, Vega V, Falcón O, Rivero JF, Torres S, León L, Fernández L, Pestano J, Díaz-Chico BN and Díaz-Chico JC (1997) Quantitative analysis of $p185^{HER\text{-}2/neu}$ protein in breast cancer and its association with other prognostic factors. *Int J Cancer* **74**: 175–179

Vindelov L and Christensen IJ (1990) An integrated set of methods for routine flow cytometric DNA analysis. *Methods Cell Biol* **33**: 127–137

Westley B and Rochefort H (1980) A secreted glycoprotein induced by estrogen in human breast cancer cell lines. *Cell* **20**: 352–362

*© Cancer Research Campaign 1999*

## References

[PDF_00535] Altman D. G., Lausen B., Sauerbrei W., Schumacher M. (1994). Dangers of using "optimal" cutpoints in the evaluation of prognostic factors.. J Natl Cancer Inst.

[PDF_00545] Capony F., Garcia M., Capdevielle J., Rougeot C., Ferrara P., Rochefort H. (1986). Purification and first characterization of the secreted and cellular 52-kDa proteins regulated by estrogens in human-breast cancer cells.. Eur J Biochem.

[PDF_00549] Capony F., Rougeot C., Montcourrier P., Cavailles V., Salazar G., Rochefort H. (1989). Increased secretion, altered processing, and glycosylation of pro-cathepsin D in human mammary cancer cells.. Cancer Res.

[PDF_00552] Christopherson W. M., Connelly P. J., Alberhasky R. C. (1983). Carcinoma of the endometrium. V. An analysis of prognosticators in patients with favorable subtypes and Stage I disease.. Cancer.

[PDF_00557] Creasman W. T., Morrow C. P., Bundy B. N., Homesley H. D., Graham J. E., Heller P. B. (1987). Surgical pathologic spread patterns of endometrial cancer. A Gynecologic Oncology Group Study.. Cancer.

[PDF_00555] Creasman W. T. (1993). Prognostic significance of hormone receptors in endometrial cancer.. Cancer.

[PDF_00560] Elangovan S., Moulton B. C. (1980). Progesterone and estrogen control of rates of synthesis of uterine cathepsin D.. J Biol Chem.

[PDF_00562] Ferrandina G., Scambia G., Bardelli F., Benedetti Panici P., Mancuso S., Messori A. (1997). Relationship between cathepsin-D content and disease-free survival in node-negative breast cancer patients: a meta-analysis.. Br J Cancer.

[PDF_00565] Foekens J. A., van Putten W. L., Portengen H., de Koning H. Y., Thirion B., Alexieva-Figusch J., Klijn J. G. (1993). Prognostic value of PS2 and cathepsin D in 710 human primary breast tumors: multivariate analysis.. J Clin Oncol.

[PDF_00569] Gion M., Mione R., Dittadi R., Romanelli M., Pappagallo L., Capitanio G., Friede U., Barbazza R., Visonà A., Dante S. (1995). Relationship between cathepsin D and other pathological and biological parameters in 1752 patients with primary breast cancer.. Eur J Cancer.

[PDF_00573] Henry J. A., McCarthy A. L., Angus B., Westley B. R., May F. E., Nicholson S., Cairns J., Harris A. L., Horne C. H. (1990). Prognostic significance of the estrogen-regulated protein, cathepsin D, in breast cancer. An immunohistochemical study.. Cancer.

[PDF_00577] Hetzel D. J., Wilson T. O., Keeney G. L., Roche P. C., Cha S. S., Podratz K. C. (1992). HER-2/neu expression: a major prognostic factor in endometrial cancer.. Gynecol Oncol.

[PDF_00589] Lotocki R. J., Copeland L. J., DePetrillo A. D., Muirhead W. (1983). Stage I endometrial adenocarcinoma: treatment results in 835 patients.. Am J Obstet Gynecol.

[PDF_00592] Lukes A. S., Kohler M. F., Pieper C. F., Kerns B. J., Bentley R., Rodriguez G. C., Soper J. T., Clarke-Pearson D. L., Bast R. C., Berchuck A. (1994). Multivariable analysis of DNA ploidy, p53, and HER-2/neu as prognostic factors in endometrial cancer.. Cancer.

[PDF_00596] Malkasian G. D., Annegers J. F., Fountain K. S. (1980). Carcinoma of the endometrium: stage I.. Am J Obstet Gynecol.

[PDF_00598] Marsigliante S., Biscozzo L., Correale M., Paradiso A., Leo G., Abbate I., Dragone C. D., Storelli C. (1994). Immunoradiometric detection of pS2 and total cathepsin D in primary breast cancer biopsies: their correlation with steroid receptors.. Br J Cancer.

[PDF_00602] Mathieu M., Rochefort H., Barenton B., Prebois C., Vignon F. (1990). Interactions of cathepsin-D and insulin-like growth factor-II (IGF-II) on the IGF-II/mannose-6-phosphate receptor in human breast cancer cells and possible consequences on mitogenic activity of IGF-II.. Mol Endocrinol.

[PDF_00606] Maudelonde T., Martinez P., Brouillet J. P., Laffargue F., Pages A., Rochefort H. (1990). Cathepsin-D in human endometrium: induction by progesterone and potential value as a tumor marker.. J Clin Endocrinol Metab.

[PDF_00609] Morisset M., Capony F., Rochefort H. (1986). The 52-kDa estrogen-induced protein secreted by MCF7 cells is a lysosomal acidic protease.. Biochem Biophys Res Commun.

[PDF_00619] Nazeer T., Church K., Amato C., Ambros R. A., Rosano T. G., Malfetano J. H., Ross J. S. (1994). Comparative quantitative immunohistochemical and immunoradiometric determinations of cathepsin D in endometrial adenocarcinoma: predictors of tumor aggressiveness.. Mod Pathol.

[PDF_00623] Newbury R., Schuerch C., Goodspeed N., Fanning J., Glidewell O., Evans M. (1990). DNA content as a prognostic factor in endometrial carcinoma.. Obstet Gynecol.

[PDF_00626] Ravdin P. M., Tandon A. K., Allred D. C., Clark G. M., Fuqua S. A., Hilsenbeck S. H., Chamness G. C., Osborne C. K. (1994). Cathepsin D by western blotting and immunohistochemistry: failure to confirm correlations with prognosis in node-negative breast cancer.. J Clin Oncol.

[PDF_00630] Ravdin P. M., de Moor C. A., Hilsenbeck S. G., Samoszuk M. K., Vendely P. M., Clark G. M. (1997). Lack of prognostic value of cathepsin D levels for predicting short term outcomes of breast cancer patients.. Cancer Lett.

[PDF_00633] Rochefort H., Capony F., Garcia M. (1990). Cathepsin D in breast cancer: from molecular and cellular biology to clinical applications.. Cancer Cells.

[PDF_00636] Roger P., Montcourrier P., Maudelonde T., Brouillet J. P., Pages A., Laffargue F., Rochefort H. (1994). Cathepsin D immunostaining in paraffin-embedded breast cancer cells and macrophages: correlation with cytosolic assay.. Hum Pathol.

[PDF_00648] Scambia G., Benedetti Panici P., Ferrandina G., Distefano M., Romanini M. E., Sica G., Mancuso S. (1995). Significance of cathepsin-D expression in uterine tumours.. Eur J Cancer.

[PDF_00656] Spyratos F., Maudelonde T., Brouillet J. P., Brunet M., Defrenne A., Andrieu C., Hacene K., Desplaces A., Rouëssé J., Rochefort H. (1989). Cathepsin D: an independent prognostic factor for metastasis of breast cancer.. Lancet.

[PDF_00660] Tandon A. K., Clark G. M., Chamness G. C., Chirgwin J. M., McGuire W. L. (1990). Cathepsin D and prognosis in breast cancer.. N Engl J Med.

[PDF_00662] Thorpe S. M., Rochefort H., Garcia M., Freiss G., Christensen I. J., Khalaf S., Paolucci F., Pau B., Rasmussen B. B., Rose C. (1989). Association between high concentrations of Mr 52,000 cathepsin D and poor prognosis in primary human breast cancer.. Cancer Res.

[PDF_00665] Touitou I., Cavaillès V., Garcia M., Defrenne A., Rochefort H. (1989). Differential regulation of cathepsin D by sex steroids in mammary cancer and uterine cells.. Mol Cell Endocrinol.

[PDF_00676] Vindeløv L., Christensen I. J. (1990). An integrated set of methods for routine flow cytometric DNA analysis.. Methods Cell Biol.

